# Supplementary material for: The methodological quality of 176,620 randomized controlled trials published between 1966 and 2018 reveals a positive trend but also an urgent need for improvement
Source: PLoS Biol. 2021 Apr 19;19(4):e3001162. doi: 10.1371/journal.pbio.3001162 (PMC8084332; doi:10.1371/journal.pbio.3001162)
Supplement: S1 Text — (DOCX) [file pbio.3001162.s001.docx]

**Supplementary Methods**

*Data collection procedures*

Step 1. Identification of all human randomized controlled trials (RCTs). The Entrez API enabled access to the PubMed database and was used via R Statistical Software using the query: "randomized controlled trial[pt] NOT (animals[mh] NOT humans[mh])". Basic information (including authors, affiliations, journal, trial registry numbers, language, and funding agency), which is indexed by the PubMed database, was downloaded.

Step 2. Filtering identified studies. Not all automatically identified studies were RCTs – despite the query in Step 1. To exclude potential contamination of the data by non-randomized, pilot and feasibility studies, a more strict selection of studies was made based on the title and abstract.

Articles were automatically excluded in the following conditions:

- When the title contained: "study protocol", "study design", "protocol for", "pilot" or "feasibility".

- When the abstract contained: "pilot study" or "feasibility study".

- When the title or abstract did not contain: "random" (in title and abstract) OR "assign" OR "allocat*" OR "placebo" OR "double-blind" (in the abstract).

- When language was other than English

Step 3. Downloading PDFs of the remaining studies after Steps 1 and 2. We used R scripts to download the PDF of each publication via the website of the respective publisher (https://github.com/wmotte/frrp). All downloaded PDFs were transformed to text data in the Extensible Markup Format (XML), using the open-source software GROBID (https://github.com/kermitt2/grobid).

Step 4. Retrieving additional data. Using the PubMed identifier (PMID), we linked the included RCTs to databases such as the Cochrane Database of Systematic Reviews (CDSR) and Scopus, where we downloaded relevant information if a PMID is provided.
