# Supplementary material for: The methodological quality of 176,620 randomized controlled trials published between 1966 and 2018 reveals a positive trend but also an urgent need for improvement
Source: PLoS Biol. 2021 Apr 19;19(4):e3001162. doi: 10.1371/journal.pbio.3001162 (PMC8084332; doi:10.1371/journal.pbio.3001162)
Supplement: S1 Table — RCT, randomized controlled trial. (DOCX) [file pbio.3001162.s002.docx]

**Supplementary Table S1.** Operationalization of variables for RCTs, authors, institutions, and journals.

| **Variable** | **Details** | |
| --- | --- | --- |
| 1. **Randomized Clinical Trial** | | |
| Risk of Bias | | Four domains of the Cochrane Risk-of-Bias tool: 1) random sequence generation, 2) allocation concealment, 3) blinding of participants and personnel, and 4) blinding of outcome assessment using validated machine learning approaches. Risk probabilities for the domains were extracted via open-source software provided by RobotReviewer. RobotReviewer is developed to score the risk of bias for four domains of the Cochrane Risk-of-Bias tool. No overall score was calculated. |
| Mention the CONSORT Statement in the publication | | Yes/No |
| Mention of RCT registration in any public database (e.g., clinicaltrials.gov) | | Yes/No |
| 1. **Author** | | |
| Gender of first and last author | | Based on the first name of an author, the API Genderize (https://genderize.io/) can determine the probability that this person is male or female. The gender with the highest probability was assigned to the author’s name. |
| The proportion of female co-authors. | | The putative gender of all authors was determined and combined into a proportion of all authors of the publication at issue. |
| Number of authors | | Continuous number. |
| Number of countries | | The total number of countries of (co-)authors. |
| Number of institutions | | The total number of institutions of (co-)authors. |
| H(irsch)-index of first and last author | | The H-index of the first and last author at the time of publication was obtained from the Scopus web portal. |
| 1. **Journal** | | |
| Medical discipline | | Categories, as downloaded from Web of Science, in Cites Journal Citation Reports, shortened list. If in multiple categories, the more specific category prevailed (e.g., cardiovascular vs general medicine, or neurology vs oncology). |
| Journal impact factor of the year before publication | | JIF was extracted for each journal from Web of Science data for the period 1997–2016 as no earlier time points were available. The JIF’s of 1997 were assigned to RCTs published before 1996. |
