# Supplementary material for: The methodological quality of 176,620 randomized controlled trials published between 1966 and 2018 reveals a positive trend but also an urgent need for improvement
Source: PLoS Biol. 2021 Apr 19;19(4):e3001162. doi: 10.1371/journal.pbio.3001162 (PMC8084332; doi:10.1371/journal.pbio.3001162)
Supplement: S2 Table — JIF, journal impact factor. (DOCX) [file pbio.3001162.s003.docx]

**Supplementary Table S2:** All journals with Journal Impact Factor (JIF) higher than 10 in the year preceding any of the individual publications in our data set.

Acta Neuropathol.

Alzheimers Dement

Am J Psychiatry

Am. J. Gastroenterol.

Am. J. Hum. Genet.

Am. J. Respir. Crit. Care Med.

Ann. Intern. Med.

Ann. Neurol.

Ann. Oncol.

Ann. Rheum. Dis.

Arch. Gen. Psychiatry

Arch. Intern. Med.

Biol. Psychiatry

Blood

BMJ

Brain

Cell

Cell Metab.

Circulation

Diabetes Care

Eur. Heart J.

Eur. Respir. J.

Eur. Urol.

Gastroenterology

Genome Biol.

Gut

Hepatology

Intensive Care Med

J. Allergy Clin. Immunol.

J. Am. Coll. Cardiol.

J. Clin. Oncol.

J. Hepatol.

J. Natl. Cancer Inst.

J. Pineal Res.

JAMA

JAMA Intern Med

JAMA Neurol

JAMA Oncol

JAMA Pediatr

JAMA Psychiatry

Lancet

Lancet Diabetes Endocrinol

Lancet Glob Health

Lancet Infect Dis

Lancet Neurol

Lancet Oncol.

Lancet Psychiatry

Leukemia

N. Engl. J. Med.

Nat. Med.

Nat. Neurosci.

Neuron

PLoS Med
