# Supplementary material for: The methodological quality of 176,620 randomized controlled trials published between 1966 and 2018 reveals a positive trend but also an urgent need for improvement
Source: PLoS Biol. 2021 Apr 19;19(4):e3001162. doi: 10.1371/journal.pbio.3001162 (PMC8084332; doi:10.1371/journal.pbio.3001162)
Supplement: S3 Table — RCT, randomized controlled trial. (DOCX) [file pbio.3001162.s004.docx]

| **RCTs** | **Risk-of-bias domain** | **25%** | **75%** |
| --- | --- | --- | --- |
| included | Allocation bias | 0.41 | 0.71 |
| excluded | Allocation bias | 0.46 | 0.72 |
| included | Randomization bias | 0.21 | 0.60 |
| excluded | Randomization bias | 0.24 | 0.61 |
| included | Blinding of people bias | 0.40 | 0.75 |
| excluded | Blinding of people bias | 0.43 | 0.77 |
| included | Blinding of outcome bias | 0.45 | 0.64 |
| excluded | Blinding of outcome bias | 0.45 | 0.65 |

**Supplementary Table S3**. Quantiles of estimated risk-of-bias domain probabilities for included and excluded RCTs.
