# Supplementary material for: The methodological quality of 176,620 randomized controlled trials published between 1966 and 2018 reveals a positive trend but also an urgent need for improvement
Source: PLoS Biol. 2021 Apr 19;19(4):e3001162. doi: 10.1371/journal.pbio.3001162 (PMC8084332; doi:10.1371/journal.pbio.3001162)
Supplement: S4 Table — RCT, randomized controlled trial. (DOCX) [file pbio.3001162.s005.docx]

| **RCTs** | **Outcome** | **Present** | **Absent** |
| --- | --- | --- | --- |
| Included | With CONSORT Statement | 11.9% | 88.1% |
| excluded | With CONSORT Statement | 8.4% | 91.6% |
| included | RCT Registration | 11.9% | 88.1% |
| excluded | RCT Registration | 8.4% | 91.6% |

**Supplementary Table S4**. Percentages for the CONSORT Statement and Registration outcomes for included and excluded RCTs.
