# Supplementary material for: The methodological quality of 176,620 randomized controlled trials published between 1966 and 2018 reveals a positive trend but also an urgent need for improvement
Source: PLoS Biol. 2021 Apr 19;19(4):e3001162. doi: 10.1371/journal.pbio.3001162 (PMC8084332; doi:10.1371/journal.pbio.3001162)
Supplement: S5 Table — (DOCX) [file pbio.3001162.s006.docx]

| Medical discipline | Domain | N | K | Low risk (CI) |
| --- | --- | --- | --- | --- |
| Anesthesiology | Randomization | 5628 | 4482 | 79.6% (78.6-80.7%) |
|  | Allocation concealment |  | 3138 | 55.8% (54.4-57.1%) |
|  | Blinding of participants and personnel |  | 2984 | 53% (51.7-54.3%) |
|  | Blinding of outcome assessment |  | 3280 | 58.3% (57-59.6%) |
| Endocrinology & metabolism | Randomization | 6080 | 2861 | 47.1% (45.8-48.3%) |
|  | Allocation concealment |  | 1634 | 26.9% (25.8-28%) |
|  | Blinding of participants and personnel |  | 2393 | 39.4% (38.1-40.6%) |
|  | Blinding of outcome assessment |  | 1818 | 29.9% (28.8-31.1%) |
| Gastroenterology & hepatology | Randomization | 3751 | 2572 | 68.6% (67.1-70%) |
|  | Allocation concealment |  | 1704 | 45.4% (43.8-47%) |
|  | Blinding of participants and personnel |  | 1468 | 39.1% (37.6-40.7%) |
|  | Blinding of outcome assessment |  | 1345 | 35.9% (34.3-37.4%) |
| General | Randomization | 11907 | 7897 | 66.3% (65.5-67.2%) |
|  | Allocation concealment |  | 5618 | 47.2% (46.3-48.1%) |
|  | Blinding of participants and personnel |  | 4479 | 37.6% (36.7-38.5%) |
|  | Blinding of outcome assessment |  | 5170 | 43.4% (42.5-44.3%) |
| Gynacology & reproduction | Randomization | 5129 | 3870 | 75.5% (74.2-76.6%) |
|  | Allocation concealment |  | 2742 | 53.5% (52.1-54.8%) |
|  | Blinding of participants and personnel |  | 1554 | 30.3% (29-31.6%) |
|  | Blinding of outcome assessment |  | 1812 | 35.3% (34-36.7%) |
| Heart & vascular | Randomization | 9218 | 4902 | 53.2% (52.2-54.2%) |
|  | Allocation concealment |  | 3050 | 33.1% (32.1-34.1%) |
|  | Blinding of participants and personnel |  | 3360 | 36.5% (35.5-37.4%) |
|  | Blinding of outcome assessment |  | 3828 | 41.5% (40.5-42.5%) |
| Immunology | Randomization | 3638 | 2140 | 58.8% (57.2-60.4%) |
|  | Allocation concealment |  | 1278 | 35.1% (33.6-36.7%) |
|  | Blinding of participants and personnel |  | 1666 | 45.8% (44.2-47.4%) |
|  | Blinding of outcome assessment |  | 1470 | 40.4% (38.8-42%) |
| Infectious | Randomization | 3931 | 2471 | 62.9% (61.3-64.4%) |
|  | Allocation concealment |  | 1600 | 40.7% (39.2-42.3%) |
|  | Blinding of participants and personnel |  | 1276 | 32.5% (31-34%) |
|  | Blinding of outcome assessment |  | 1316 | 33.5% (32-35%) |
| Neurology | Randomization | 5269 | 3257 | 61.8% (60.5-63.1%) |
|  | Allocation concealment |  | 1977 | 37.5% (36.2-38.8%) |
|  | Blinding of participants and personnel |  | 2109 | 40% (38.7-41.4%) |
|  | Blinding of outcome assessment |  | 2330 | 44.2% (42.9-45.6%) |
| Oncology | Randomization | 6980 | 4392 | 62.9% (61.8-64.1%) |
|  | Allocation concealment |  | 2947 | 42.2% (41.1-43.4%) |
|  | Blinding of participants and personnel |  | 1535 | 22% (21-23%) |
|  | Blinding of outcome assessment |  | 1575 | 22.6% (21.6-23.6%) |
| Other | Randomization | 43051 | 25954 | 60.3% (59.8-60.7%) |
|  | Allocation concealment |  | 14775 | 34.3% (33.9-34.8%) |
|  | Blinding of participants and personnel |  | 11507 | 26.7% (26.3-27.2%) |
|  | Blinding of outcome assessment |  | 15432 | 35.8% (35.4-36.3%) |
| Pediatrics | Randomization | 2819 | 1945 | 69% (67.2-70.7%) |
|  | Allocation concealment |  | 1474 | 52.3% (50.4-54.1%) |
|  | Blinding of participants and personnel |  | 737 | 26.1% (24.5-27.8%) |
|  | Blinding of outcome assessment |  | 1234 | 43.8% (41.9-45.6%) |
| Psychiatry | Randomization | 7429 | 4277 | 57.6% (56.4-58.7%) |
|  | Allocation concealment |  | 2155 | 29% (28-30.1%) |
|  | Blinding of participants and personnel |  | 2986 | 40.2% (39.1-41.3%) |
|  | Blinding of outcome assessment |  | 3017 | 40.6% (39.5-41.7%) |
| Surgery | Randomization | 6912 | 4584 | 66.3% (65.2-67.4%) |
|  | Allocation concealment |  | 2792 | 40.4% (39.2-41.6%) |
|  | Blinding of participants and personnel |  | 1705 | 24.7% (23.7-25.7%) |
|  | Blinding of outcome assessment |  | 2455 | 35.5% (34.4-36.7%) |
| Urology & nephrology | Randomization | 3460 | 1935 | 55.9% (54.3-57.6%) |
|  | Allocation concealment |  | 996 | 28.8% (27.3-30.3%) |
|  | Blinding of participants and personnel |  | 1091 | 31.5% (30-33.1%) |
|  | Blinding of outcome assessment |  | 742 | 21.4% (20.1-22.9%) |

**Supplementary Table S5**. Total number (N) of trials published in the period 2005–2018 in the different medical disciplines with the number (K) and corresponding proportion (percentage with 95% confidence interval) of trials with a risk-of-bias probability below 50% (i.e., ‘low risk’).
