# Supplementary figures and images for: The methodological quality of 176,620 randomized controlled trials published between 1966 and 2018 reveals a positive trend but also an urgent need for improvement
Source: PLoS Biol. 2021 Apr 19;19(4):e3001162. doi: 10.1371/journal.pbio.3001162 (PMC8084332; doi:10.1371/journal.pbio.3001162)

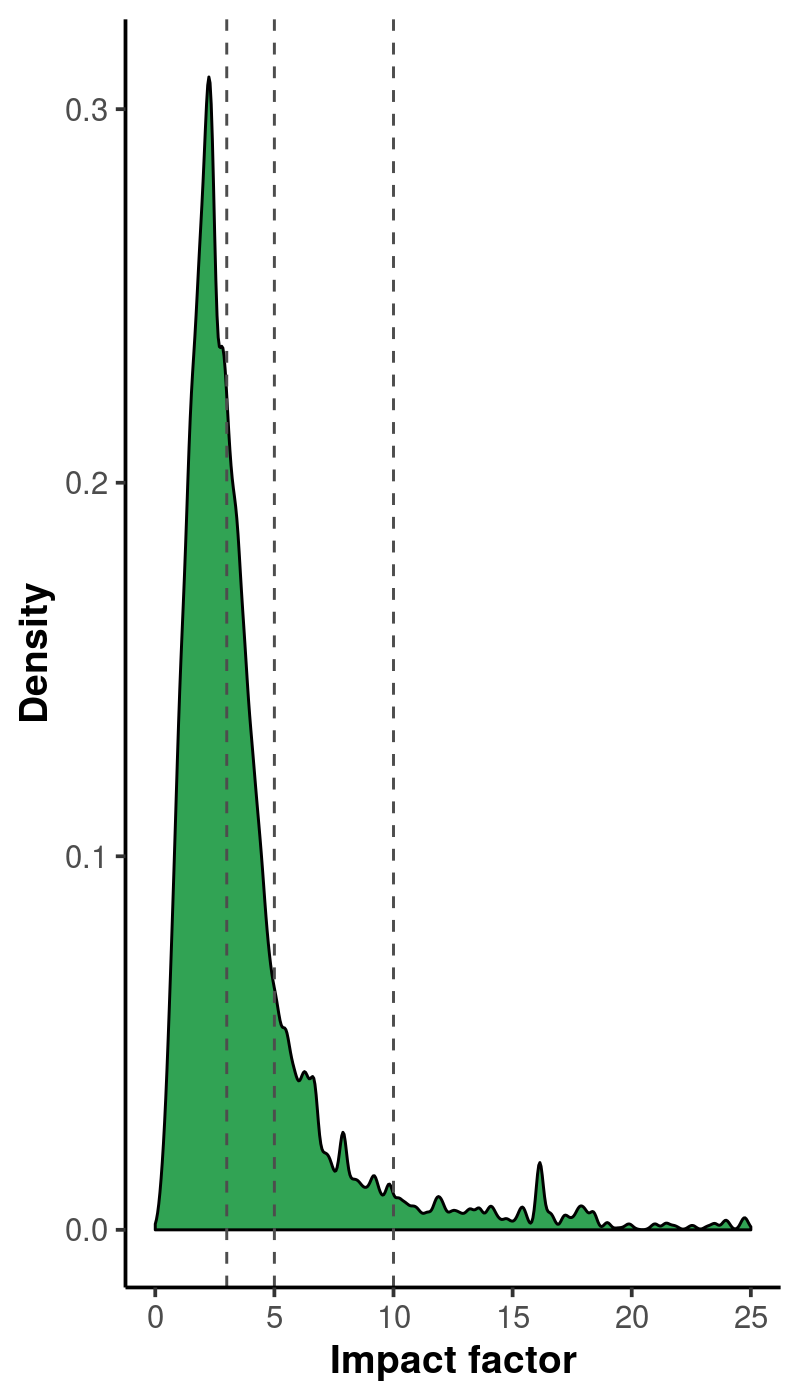

Supplement: S1 Fig — The JIF of a journal in the year following the publication date of the RCT was used. Density represents the probability of a trial to belong to a given impact factor. JIF, journal impact factor; RCT, randomized controlled trial. (TIFF) [file pbio.3001162.s007.tiff]

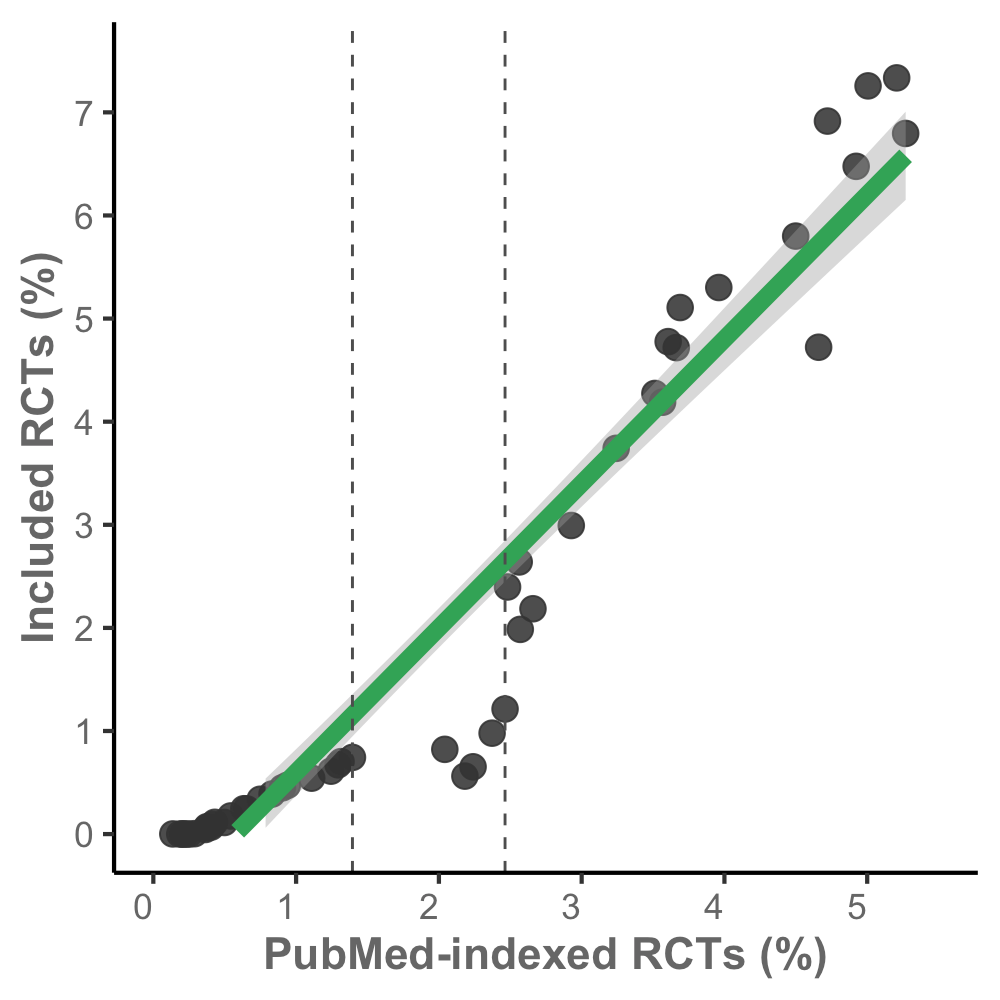

Supplement: S2 Fig — The year 1993 and 1998 are marked with vertical dashed lines. RCT, randomized controlled trial. (TIFF) [file pbio.3001162.s008.tiff]

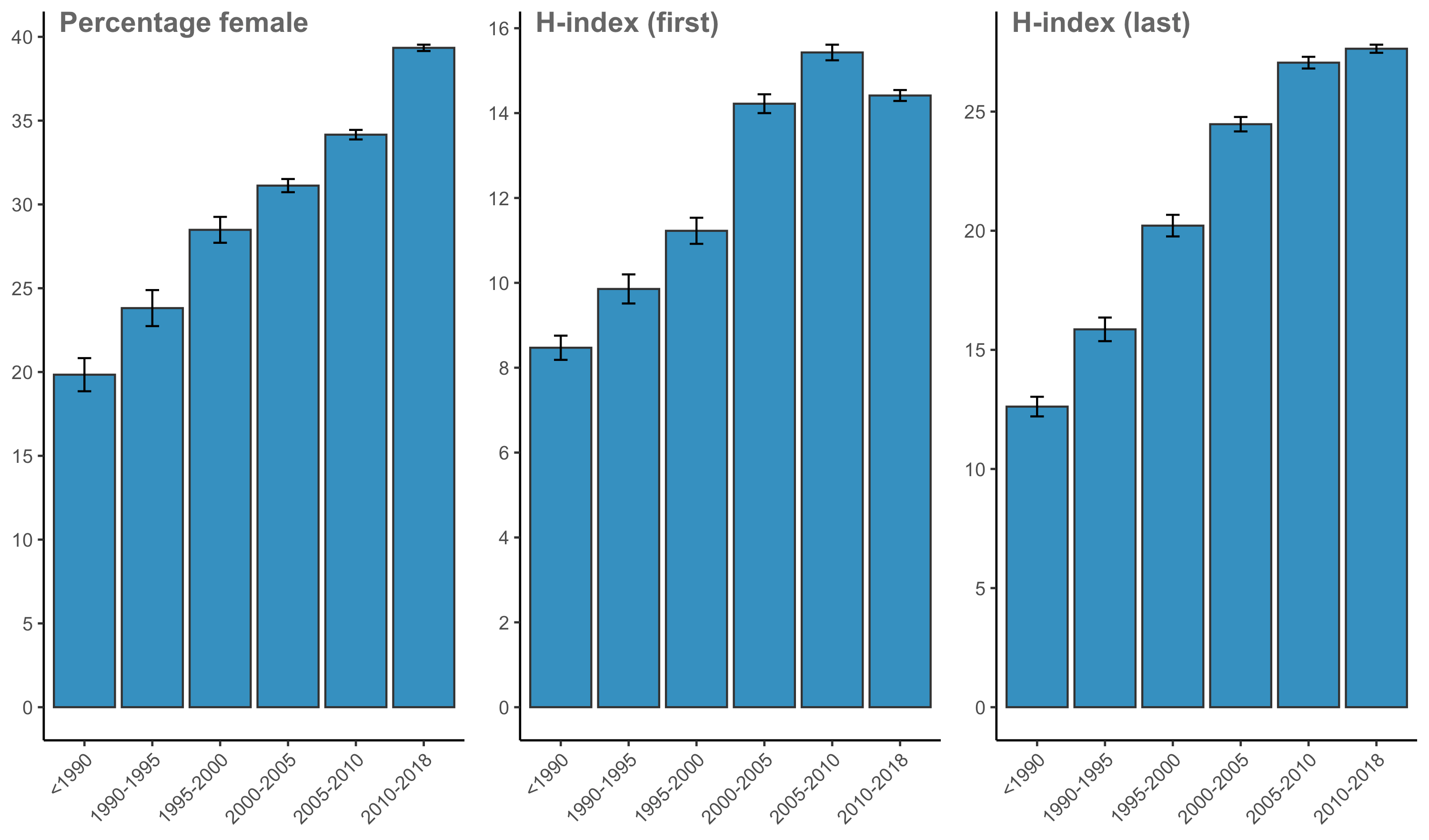

Supplement: S3 Fig — RCT, randomized controlled trial. (TIFF) [file pbio.3001162.s009.tiff]

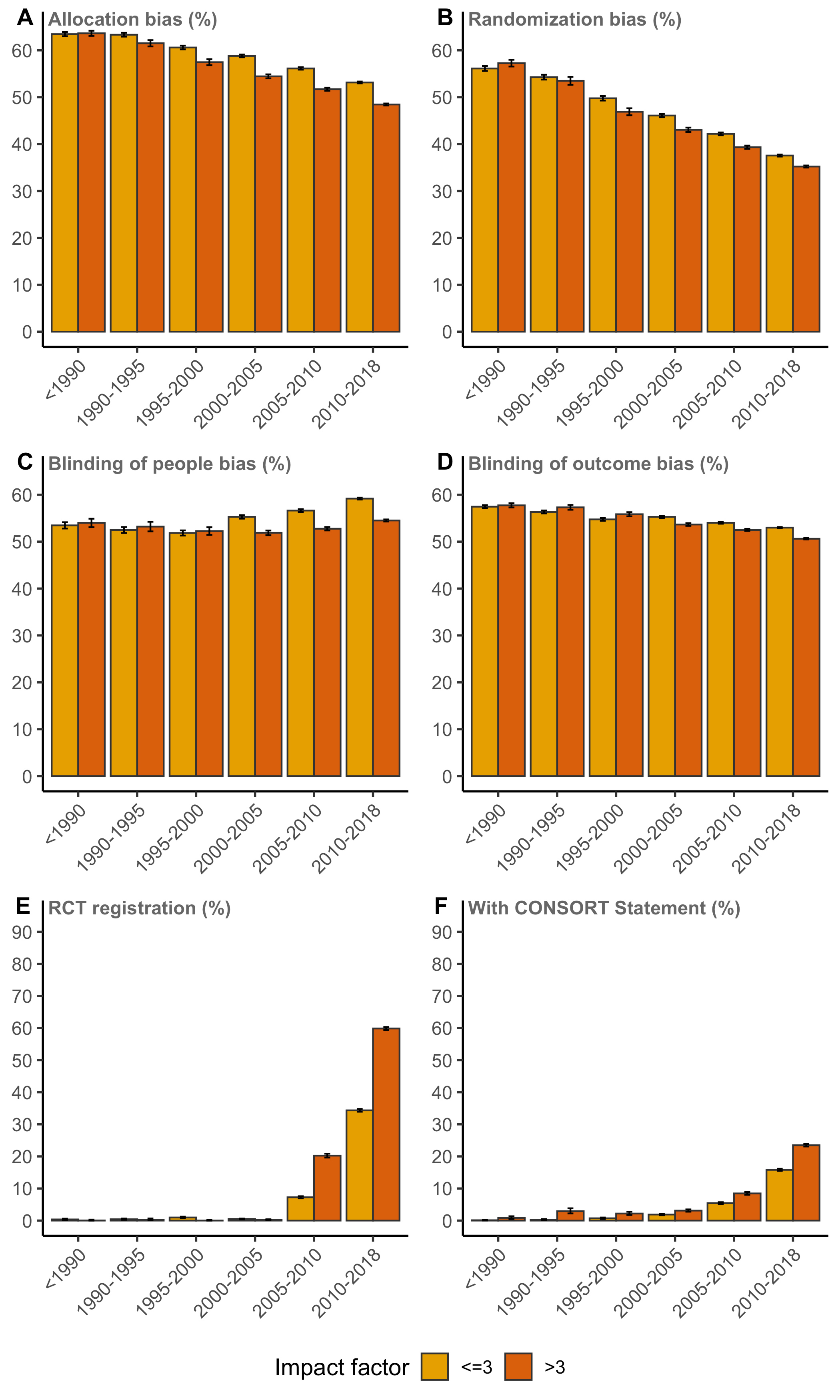

Supplement: S4 Fig — Risk of bias due to inadequate allocation concealment (A), random sequence generation bias (B), the bias in blinding of patients and personnel (people) (C), the bias in blinding of outcome assessment (D), RCT registration (E), and mentioning of the CONSORT Statement (F) plotted over time for RCTs published in journals with JIF >3 and journals with JIF <3. The indicated stratum range is up to but not including the last year. JIF, journal impact factor; RCT, randomized controlled trial. (TIFF) [file pbio.3001162.s010.tiff]

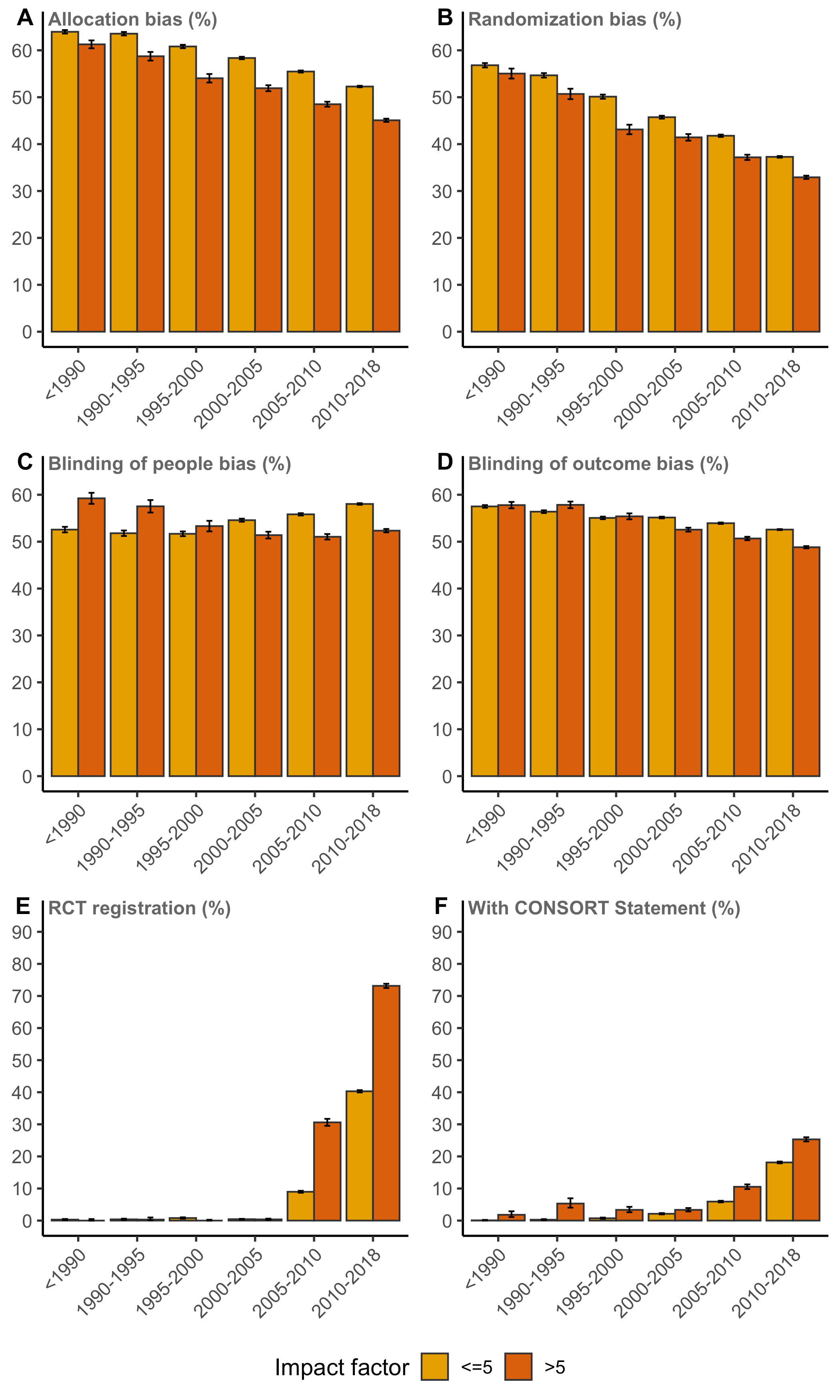

Supplement: S5 Fig — Risk of bias in allocation concealment (A), the bias in randomization (B), the bias in blinding of patients and personnel (people) (C), the bias in blinding of outcome assessment (D), RCT registration (E), and mentioning of the CONSORT Statement (F) plotted over time for RCTs published in journals with JIF >5 and journals with JIF <5. The indicated stratum range is up to but not including the last year. JIF, journal impact factor; RCT, randomized controlled trial. (TIFF) [file pbio.3001162.s011.tiff]

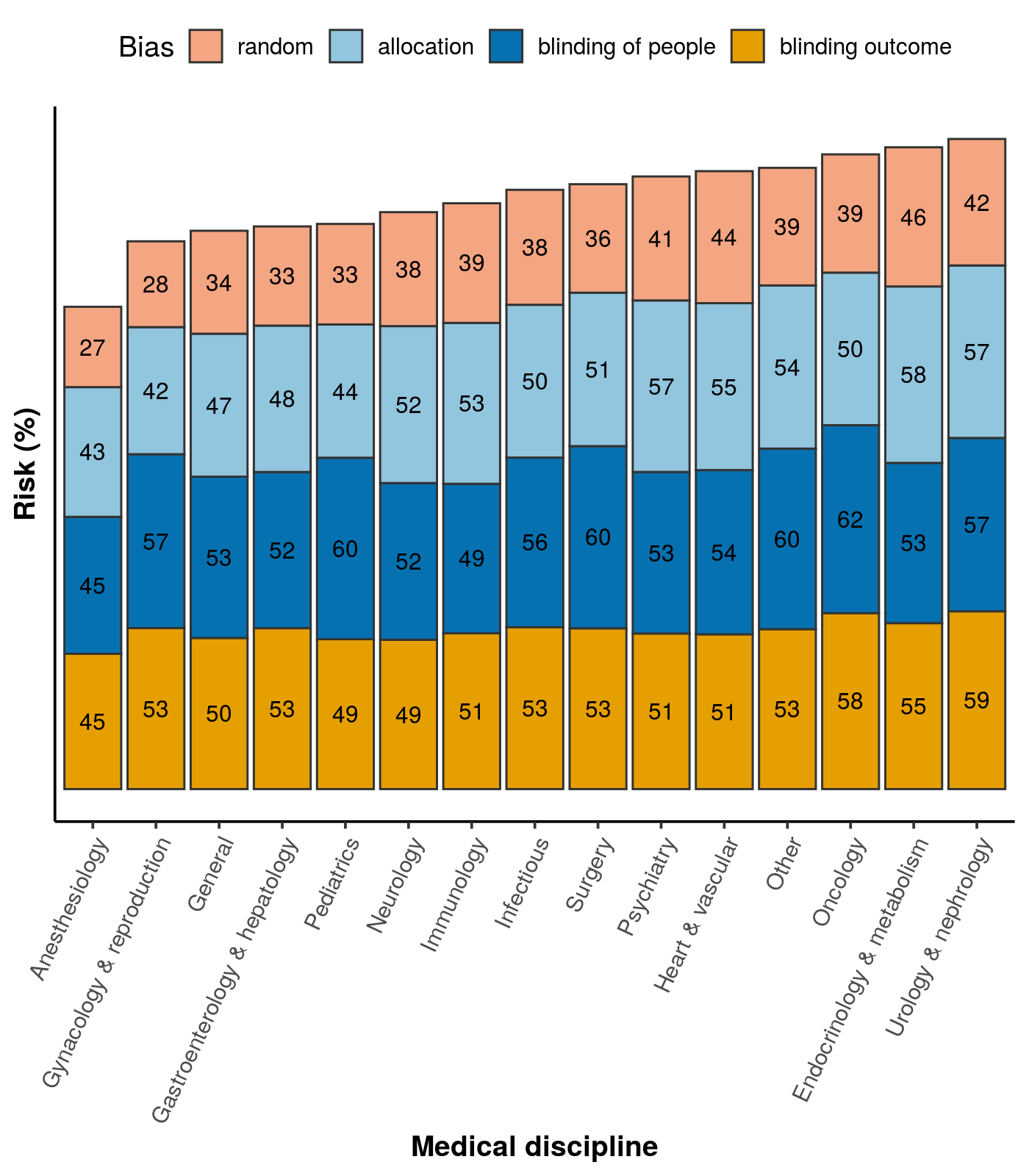

Supplement: S6 Fig — “random”: bias in randomization; “allocation”: bias in allocation concealment; “blinding of people”: bias in blinding of patients and personnel; “blinding outcome”: bias in the blinding of outcome assessment. (TIFF) [file pbio.3001162.s012.tiff]

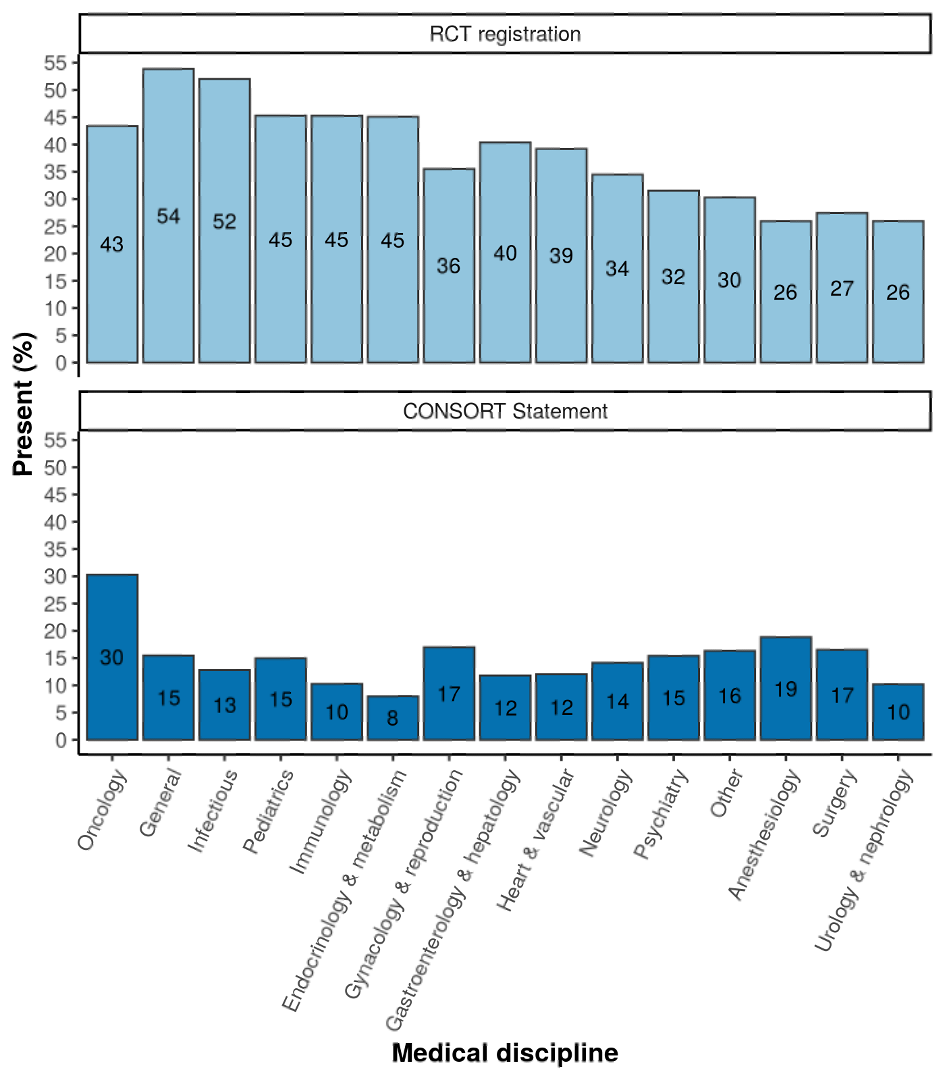

Supplement: S7 Fig — RCT, randomized controlled trial. (TIFF) [file pbio.3001162.s013.tiff]

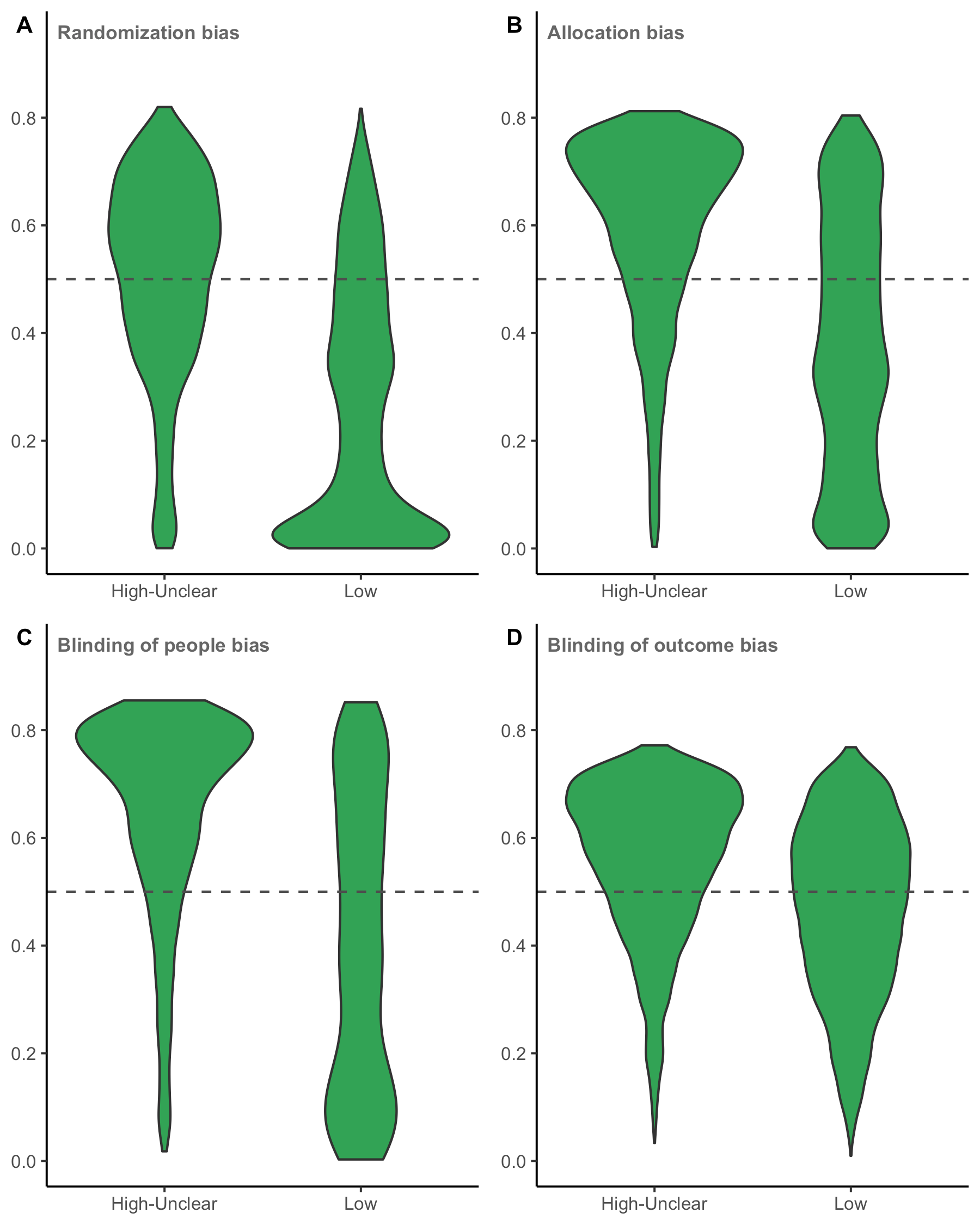

Supplement: S8 Fig — RCT, randomized controlled trial. (TIFF) [file pbio.3001162.s014.tiff]
