# Supplementary material for: The methodological quality of 176,620 randomized controlled trials published between 1966 and 2018 reveals a positive trend but also an urgent need for improvement
Source: PLoS Biol. 2021 Apr 19;19(4):e3001162. doi: 10.1371/journal.pbio.3001162 (PMC8084332; doi:10.1371/journal.pbio.3001162)
Supplement: Attachment — Submitted filename: PLOS_Biol_rebuttal_final.pdf [file pbio.3001162.s015.pdf]

<u>Academic Editor</u>

We would like to thank the editor for his constructive comments and summary of the reviewers. We have addressed all comments and have indicated how changes have been made.

1. *After reading the comments made by our reviewers, we would like to invite you resubmit your paper in which you address these. In particular, I encourage you to expand the manuscript somewhat to address the issue of selection of input. This selection comes with the methodology and is thus is inevitable, but understanding its effect in more depth is also pivotal in understanding the results. Through your methods you have indeed "seen" more papers than is possible with the more traditional hand work. Some extra digging to understand how problematic this selection is, is very much welcome. Some ideas to provide this insight could be:*

- get some insight into missing data: what years/location/ type of journal etc where these missing papers?

- perform some sensitivity analyses to get a feeling of the impact of this selection on the results: worst case/best case scenarios, or even more sophisticated methods are welcome or even preferred

I truly hope that you will be able to provide some extra quantified insights and not just leave it to an academic discussion based on hypothetical. The reason for this is this issue is not only fundamental to this particular paper, but to the whole approach of using machines instead of hands/eyes/brains in meta-research on reporting of scientific results.

Answer: We thank the editor for the constructive assessments and helpful suggestions which we fully agree with. Specifically, we have performed additional analyses regarding missing data. Most RCTs are not included as we do not have access to full-text data. This makes it difficult to make definitive conclusions on the missing data with regard to risk of bias, CONSORT statement and trial registration. However, we did exclude RCT publications with an uncertain year of publication (7,307). Therefore, we compared these 7,307 RCT with the included 176,620 RCTs. The distribution of the risk-of-bias domain probabilities of included and excluded RCTs were comparable, with very similar interquartile ranges (see Suppl. Table S2). Moreover, the presence of the 'With CONSORT Statement' and 'RCT Registration' outcomes were 3–4% lower in the excluded RCTs (Suppl. Table S3). Moreover, the full text sample size was mostly in line with the overall number of potential RCTs in the PubMed database (Suppl. Figure S6). Generally, the % of included RCTs was stable over time, except for the time period between 1993 and 1998 when the library switched from scanned versions to online subscriptions, potentially lowering the yield of automated full text downloads. This period is before the release and implementation of the discussed guidelines, we think our sample still maps the important patterns in the overall RCT population. We have added these results to the manuscript and mentioned the limitation in the Discussion.

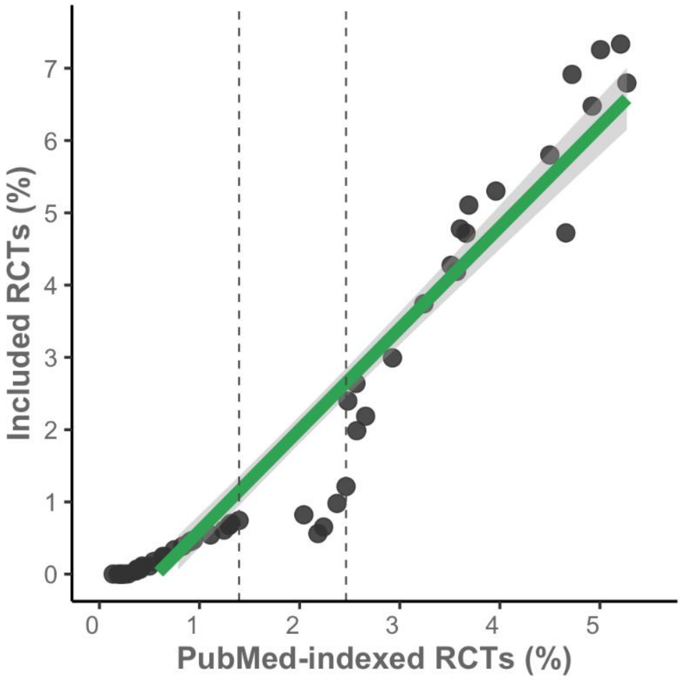

Supplementary Figure S6. *The number of included RCTs against the total number of RCTs indexed in the PubMed database for the study period (1966–2018). The year 1993 and 1998 are marked with vertical dashed lines.*

Moreover, we have now validated our predictions with a newly designed validation dataset comprised of human expert ratings of RCTs. This has allowed us to determine the robustness of our results, including which RoB domain score is best and which is worst. Our manual validation based on 63,327 extracted and standardized human risk-of-bias assessments, as published in a Cochrane Database of Systematic Reviews showed accuracies of over 70% and kappa values between 0.25–0.43. Even though this is not perfect, it should be borne in mind that agreement between human reviewers is often not much higher. For example, based on 376 RCTs overall kappa values of interrater risk-of-bias scores ranged from 0.40–0.42, although some domains were clearly higher, such as random sequence bias agreements between human raters (kappa 95% CI: 0.56–0.71).

Finally, we present our quantitative data as proportions over time with 95% confidence intervals. Given the current tendency to switch from null-hypothesis testing to output presentation, we have not emphasized P the formal testing results too much. Given the large sample size all trend analyses are highly significant with P values < 0.001, both for overall trend fits as well as individual year-category comparisons.

Together, we feel we have addressed the majority of the constructive comments of the reviewer as summarized by the Academic Editor.

2. *I applaud your efforts to make your research as open as possible - github/osf. When looking at the OSF preregistered protocol, some major elements deviate or are missing. Some examples that just caught my eye are: "prediction" as the main aim in protocol vs emphasis on "over time" in manuscript; "main difference between included and excluded trials will be*

described" in protocol (see comments earlier; consort: mentioning consort vs using statreviewer; description of excluded data and imputation of missing data (in line with earlier comments). We understand that plans and the final result do not always line up, but there has to be a substantial overlap and explanation of any non-overlap to make sense for the reader. We encourage you to add this information to the manuscript or its supplemental information. If the current ms is more a description of the dataset and the "prediction" element will be described in a separate paper, please explain, and remove the reference to the OSF protocol. On a similar note, I encourage you to highlight substantial additions or changes to your methods during this peer review as such in the new version of the manuscript. This way, there is a continuous line between the preregistered protocol on OSF and the actual reporting in this manuscript. It also shows readers/prospective peer reviewers the added value of the peer review process.

Answer: We thank the editor for pointing this out. The protocol that we refer to is not the protocol for the current paper, although it does belong to the same project. The protocol refers to a prediction paper that is currently being carried out. Methodology does overlap, but is at some places different. Therefore, we agree that referral to this protocol as it currently is, may be confusing.

We have therefore changed the text to: *"A protocol for a prediction paper was registered prior to study conduct [1]. This protocol does not apply to the current manuscript which is a description of the dataset used in the protocol. Nevertheless, it does contain details on methodology that we used in this manuscript. The database and scripts are available through GitHub (see Data Sharing), and the results are disseminated through the medRxiv pre-print server. "*

===

Reviewer #1:

We would like to thank the reviewer for his/her constructive comments. We have addressed each comment and have indicated how changes have been incorporated in our revised manuscript.

1. *This manuscript aims to report an analysis of the evolution over time of publication characteristics, risk of bias and quality of reporting of randomized controlled trials (RCTs). It is based on a very large sample of RCTS analyzed with the help of machine learning tools. This is, to date, the largest mapping of RCTs. The manuscript is overall clear and well written. The topic is interesting, although not completely original regarding the evolution of risk of bias over time. I was slightly surprised by the choice of Plos Biology to submit this manuscript. Although Plos Biology is very interested in transparency and good methodological standards, I am not sure this journal publishes a lot of RCTs.*

Answer: We would like to thank the reviewer for the positive comments. As PLOS Biology has a specific meta-research section which explicitly invites authors to submit manuscripts on this topic [2], we think our clinically-oriented meta-research manuscript does fit this section as it describes characteristics and biases on quite a large set of RCTs.

2. *The objective should be well-formulated in particular I would be more precise at the end of the introduction section.*

Answer: We thank the reviewer for the suggestion. We now write at the end of the Introduction:

"Therefore, this study aimed to provide a comprehensive and unbiased analysis of developments in the clinical trial landscape between 1966 and 2018 based on 176,620 full-text RCT publications. Specifically, we identified full-text RCTs via Pubmed and then used automated analyses to assess Risk of Bias, CONSORT statement use, trial registration, and characteristics related to publication (number of authors, journal impact factor, medical discipline)."

3. *A key question is the validity of the automated tools used to evaluate characteristics (for example author gender) or risk of bias. We need more elements to be sure that these tools work well. Regarding risk of bias, the authors indicate an agreement between humans and Robot Reviewer ranging from 39% to 91%. I did not find that very similar to the agreement between humans and 39% is a low rate. Did the authors conduct some manual verifications? The authors report that they link their data with Cochrane data. A validation for risk of bias could be the comparison between risk of bias assessment obtained from the automated tool with corresponding risk of bias reported in Cochrane reviews.*

Answer: This is an excellent suggestion. We have now validated our predictions with a newly designed validation dataset comprised of human expert ratings of RCTs. This has allowed us to determine the robustness of our results, including which risk-of-bias score is overall best and which is worst. Our manual validation based on 63,327 extracted and standardized human risk-of-bias assessments, as published in a Cochrane Database of Systematic Reviews showed accuracies of over 70% and kappa values between 0.25–0.43. Even though this sounds far from impressive, it should be borne in mind that agreement between human reviewers is often not much higher. For example, based on 376 RCTs overall kappa values of interrater risk-of-bias scores ranged from 0.40–0.42, although some domains were clearly higher, such as random sequence bias agreements between human raters (kappa 95% CI: 0.56–0.71).

We now write in the Methods section:

"Risk-of-bias assessment validation
To determine the accuracy of the automated risk of bias assignment to RCTs in our database, we validated a large subset of RCTs with human assessments obtained from systematic reviews. To this end, we inspected all PMIDs of RCTs included in the 8,075 systematic reviews (Issue 6, 2020) published in the Cochrane Database of Systematic Reviews (CDSR). The CDSR is part of the international charitable Cochrane organization, with more than fifty review groups based at research institutions worldwide, aiming to facilitate evidence-based choices about health interventions. Contributions to the database come from these review groups as well as from ad hoc teams. The systematic reviews in the CDSR were identified through PubMed, for the period to 2000 and 2020. This was done with the NCBI's EUtils API with the following query: "Cochrane Database Syst Rev"[journal] AND ("2000/01/01"[PDAT]: "2020/05/31"[PDAT])". We limited assessment of the latest systematic review updates as to prevent overlap between included RCTs. Review protocols were excluded.

All systematic review tables containing the words 'bias' and 'risk' were inspected on human export risk-of-bias text associated with a PMID. If a PMID matched with a PMID in our full-text database and the risk of bias domain concerned 'allocation concealment', 'random

sequence generation', 'blinding of participants and personnel' or 'blinding of the outcome', the human risk-of-bias text was extracted.

Since the risk-of-bias data reporting in the CDSR is not imposed or standardized, we, therefore, manually inspected all human risk-of-bias texts and assigned the judgment to the correct and standardized risk-of-bias category. Judgments were binarized into 'low' and 'high/unclear' risk of bias and used to validate our automated binarized risk-of-bias probabilities (i.e., <= 0.5 'low' risk, and > 0.5 'high/unclear' risk) in terms of accuracy, sensitivity, specificity, and kappa."

And in the Results section:

"Risk-of-bias assessment validation
In total, 63,327 *matching human risk-of-bias judgments and automated risk-of-bias predictions were extracted from 17,394 unique RCTs included in a systematic review (**Table 1**). Overall, automated accuracy to determine 'randomization bias', 'allocation bias' and 'blinding of people bias' was above 70%. The 'blinding of outcome bias' had a lower accuracy, namely 63.3%. The distribution of risk-of-bias predictions are shown in **Supplementary Figure S5**.*

*We also classified the chance-corrected level of agreement in terms of kappa. Kappa ranges from: 'poor' (<0.00), 'slight' (0.00–0.20), 'fair' (0.21–0.40), 'moderate' (0.41–0.60), 'substantial' (0.61–0.80), to 'almost perfect' (0.81–1.0). Within this scale, moderate agreement was found for randomization and blinding of people bias and fair agreement was found for allocation bias and blinding of outcome bias (**Table 1**)."*

And in the Discussion:

"Our manual validation based on 63,327 *extracted and standardized human risk-of-bias assessments, as published in a Cochrane Database of Systematic Reviews showed accuracies of over 70% and kappa values between 0.25–0.43. Even though this sounds far from impressive, it should be borne in mind that agreement between human reviewers is often not much higher. For example, based on 376 RCTs overall kappa values of interrater risk-of-bias scores ranged from 0.40–0.42, although some domains were clearly higher, such as random sequence bias agreements between human raters (kappa 95% CI: 0.56–0.71)."*

4. *Regarding risk of bias, it is not fully detailed how it works. It seems that the result is rendered as a probability but this is not clear enough. Is it a probability of being at high risk of bias for the domain from 0 to 100%? Usually, the terms score is not adapted to risk of bias.*

Answer: The probability below 0.5 corresponds to the 'low bias' assessments. Probabilities above 0.5 to 'high/unclear' judgements. We have also used this threshold to binarize our probabilities in the validation characterization. We have kept the probabilities in the other plots to prevent information loss due to binarization.

We have now specified this in the Methods section:
"The machine-learning output is a probability, with values below 0.5 corresponding with 'low risk', and above 0.5 with 'high or unclear' risk of bias."

5. *Did the authors exclude trials presenting some particularities for which risk of bias assessment needs to be adapted? For example cluster or cross-over trials?*

Answer: We did not specifically exclude RCTs with detailed methodologies. Even though the reviewer raises a fair point, an exhaustive list of particularities would be difficult to achieve. For example, any RCT publication containing the word "cross-over" was less than 1% of the total of RCTs, and exclusion of these RCTs did not essentially change any of the results. We therefore abstained from excluding specific queries as not to introduce bias.

Nevertheless, we have performed additional analyses regarding missing RCTs. Most RCTs are not included as we do not have access to full-text data. This makes it difficult to make definitive conclusions on the missing data with regard to risk of bias, CONSORT statement and trial registration. However, we did exclude RCT publications with an uncertain year of publication (7,307). Therefore, we compared these 7,307 RCT with the included 176,620 RCTs. The distribution of the risk-of-bias domain probabilities of included and excluded RCTs were comparable, with very similar interquartile ranges (see Suppl. Table S2). Moreover, the presence of the 'With CONSORT Statement' and 'RCT Registration' outcomes were 3–4% lower in the excluded RCTs (Suppl. Table S3). Moreover, the full text sample size was generally in line with the overall number of potential RCTs in the PubMed database (Suppl. Figure S6). Generally, the % of included RCTs was stable over time, except for the time period between 1993 and 1998 when the library switched from scanned versions to online subscriptions, potentially lowering the yield of automated full text downloads. this period is before the release and implementation of the discussed guidelines, we think our sample still maps the important patterns in the overall RCT population. We have added these results to the manuscript and mentioned the limitation in the Discussion.

To address these relevant issues, we have included the additional analyses in the Methods and Results section. In our discussion, we now write:
"First, our selection of RCTs may have been biased as full-text RCTs may not have been missing at random (e.g., with particular year of publication or RCT methodology). Most RCTs are not included as we do not have access to full-text data, and this makes it difficult to make definitive conclusions on the missing RCTs with regard to risk of bias, CONSORT statement use, and trial registration. However, an additional analysis of RCTs with an uncertain year of publication (7,307) that were excluded yielded similar distribution of the risk-of-bias domain probabilities. Moreover, the included RCTs in our analyses were generally in line with the overall number of potential RCTs in the PubMed database (Suppl. Figure S6), except for the time period between 1993 and 1998 when libraries switched from scanned versions to online subscriptions, potentially lowering the yield of automated full text downloads. However, this period is before the release and implementation of the discussed guidelines, and our sample still maps the important patterns in the overall RCT publications over time."

6. *The authors evaluated random sequence generation, allocation concealment, blinding of patients and personnel and blinding of outcome assessment as key domains for risk of bias. I did not understand why they did not consider incomplete outcome data as it is another key domain. The authors seem to link this domain with publication bias (methods section, page 7, line 132) or the sentence is unclear?*

Answer: This is an excellent point. However, incomplete data assessment is currently not assessed in our machine-learning model, and we therefore could not quantify it.

We write in the Discussion:
"Incomplete outcome data was not assessed as domain for risk of bias in the current manuscript as it is not assessed in our automated procedure."

"Third, we did not investigate all aspects of methodological rigor. In our study, we could not check for forms of attrition bias (e.g., incomplete outcome data) or reporting bias (e.g., selective outcome reporting), which both would require a direct comparison between the trial registration and actual trial publication report."

7. *It seems from the statistical analysis that the authors evaluated the statistical significance of the comparisons between 1990-1995 and 2010-2018. Why that? A trend test considering all strata may be more appropriate.*

Answer: This is a great suggestion. We already included several trend statistics, but have added some of those more explicitly where relevant. These include:

- *The risk of bias due to not blinding outcome assessment decreased over time, from 56.6 (56.4–56.9%) to 51.8 (51.7–51.9%; P< 0.001 for overall trend;* ***Figure 3D****).*
- *There was a relatively stronger decrease in the risk of bias due to non-random sequence generation, from 54.0% (CI: 53.6–54.5%) for trials in 1990–1995 to 36.4% (36.3–36.6%; P<0.001 for overall trend).*

8. *I think there are (too) many results in this study. The authors should be sure to be consistent between objectives and results presented. For example, in the abstract, the objective does not mention risk of bias. Some results are less important. It is interesting to evaluate the trends in numbers of authors, institutions, gender but why the use of positive and negative words. The list of positive, negative and neutral words is probably not exhaustive.*

Answer: We agree with the reviewer and have removed the list of positive, negative, and neutral words, and moved the percentage of female authors, and the use of H-index to the supplements. In the abstract, we explicitly mention Risk of Bias as part of the objectives, and now write: *"We therefore mapped trends over time in risk of bias, trial registration, reporting according to CONSORT, and characteristics of publication and authors."*

9. *The term 'quality' is very criticized. I would refer to risk of bias or at a minimum methodological quality.*

Answer: We agree with the reviewer and have changed the manuscript accordingly. Specifically, we have also changed the title to reflect this valuable suggestion.

10. *Some elements are not clear in particular the start date for this study. In the abstract, it seems 1966. But it appears from the method that there is only one stratum before 1990 because there were relatively few trials before 1990. Does it mean that this stratum includes trials from 1966 to 1990? In the flow chart, it rather seems that the authors excluded RCTs published before 1988. Why? It is not mentioned in the methods. Please clarify.*

Answer: We apologize for this incorrect statement in our flow chart. We included 5,108 RCTs published before 1988 in our analyses, as far back to 1966. The flow chart is now corrected.

11. *Minor comments*

- *In the abstract, introduction and discussion, the authors should be careful regarding the wording used. For example, in the abstract: "responsible research practices and reporting guidelines are increasingly important but whether these efforts have improved RCT quality is unknown. The same in Introduction section: "In other words, have these initiatives and measures improved the quality, transparency and reproducibility of RCTS. The objective of this article is to evaluate the evolution over time. Whether improvements over time are related to the development and use of reporting guidelines is another question that cannot be answered with this study design.*

Answer: We agree with the reviewer: we cannot infer causality from our analyses, and have changes the text accordingly in the abstract, introduction, and discussion.

- *This is not the "use" of CONSORT, this is the "reporting" of CONSORT in text. I think that many RCTS may be compliant with the CONSORT without mentioning the name.*

Answer: This is a fair point which we have changed now.

- *Introduction: First sentence of the second paragraph, instead of "For a longer time period", I would give the date of first publication of the CONSORT. I would not have presented CONSORT and trial registration in the same sentence as this suggests a link between them.*

Answer: We have changed this and now write: *"Already in 1996, CONSORT criteria have been introduced to improve RCT reporting [3]. Moreover, mandatory RCT registration by the International Committee of Medical Journal Editors (ICMJE) has been put forward [4,5]."*

- *In the methods section, the authors report search of Medline, should be added "via PubMed" after to be consistent with other parts of the manuscript*

Answer: We have changed this.

- *When mentioning trial registration, it should be interesting to give the number of trial registration from 2005 and 2010. The same for CONSORT from 2000.*

Answer: We have added these numbers to the main text.

- *Page 14, line 230: not clear to what refers the p-value reported here?*

Answer: We have now clarified this in the manuscript: *"This also applied to randomization and blinding of participants and personnel and outcome assessment, even though the results were less pronounced compared to allocation concealment bias (P<0.0001, all domains, for latest time point; **Figure 4B-D**)."*

==

Reviewer #2: [identifies himself as Rustam Al-Shahi Salman]
I congratulate the authors for completing an enormous and impactful body of work. I have no suggestions for improvement.

Answer: We would like to thank Dr. Al-Shahi Salman for his positive assessment.

==

Reviewer #3:
This is a meta-research study investigating the evolution of the quality of reporting and key methods of randomized controlled trials (RCTs) using meta-data from a huge number of published RCTs. To handle such an impressive number of articles (> 175,000), the author relied on the use of machine learning (ML) algorithms to extract relevant data. The sample size is a major asset of the present study compared to many other meta-research studies, that have been hampered by much lower sample sizes. But there are some limitations, too, that could be better taken into account or better emphasized.

Answer: We would like to thank the reviewer for his/her constructive comments. We have addressed each comment and have indicated how changes have been incorporated in our revised manuscript.

1. *122 810 RCTs were excluded for technical reasons (no institutional license, failing to download full text ...), which represents almost 70% of the number included. In an RCT or an epidemiological study, it would have been considered poor methodology to exclude 41% of the sample, and this deserves discussion. I do not claim that the results of the study are not worthwhile or more biased than other meta-research studies that have often used additional eligibility criteria (e.g. by focusing on a more limited range of journals, for instance). But again, we expect some extreme rigor for such type of studies, and the choices leading to the analyzed sample size and their possible impact on the results should be thoroughly discussed.*

Answer: Most RCTs are not included as we do not have access to full-text data. This makes it difficult to make definitive conclusions on the missing data with regard to risk of bias, CONSORT statement and trial registration. However, we did exclude RCT publications with an uncertain year of publication (7,307). Therefore, we compared these 7,307 RCT with the included 176,620 RCTs. The distribution of the risk-of-bias domain probabilities of included and excluded RCTs were comparable, with very similar interquartile ranges (see Suppl. Table S2). Moreover, the presence of the 'With CONSORT Statement' and 'RCT Registration'

outcomes were 3–4% lower in the excluded RCTs (Suppl. Table S3). Moreover, the full text sample size was generally in line with the overall number of potential RCTs in the PubMed database (Suppl. Figure S6). Generally, the % of included RCTs was stable over time, except for the time period between 1993 and 1998 when the library switched from scanned versions to online subscriptions, potentially lowering the yield of automated full text downloads. this period is before the release and implementation of the discussed guidelines, we think our sample still maps the important patterns in the overall RCT population. We have added these results to the manuscript and mentioned the limitation in the Discussion.

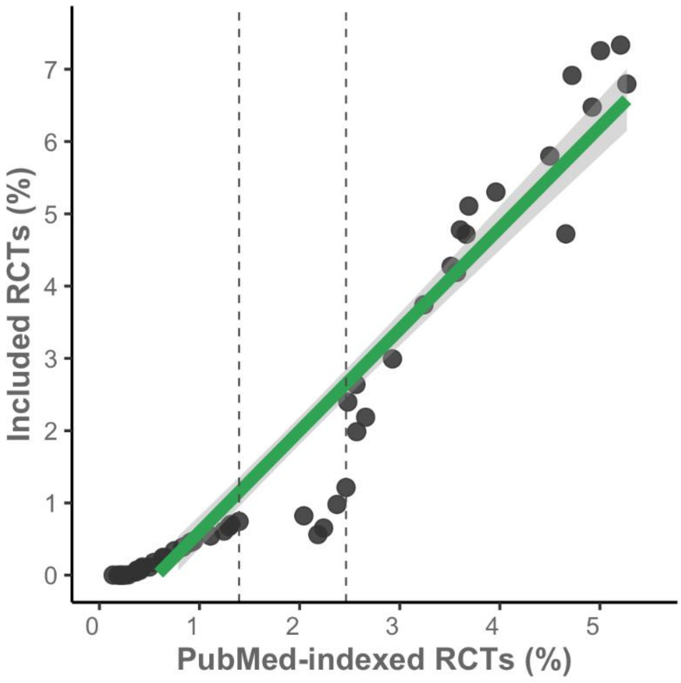

Supplementary Figure S6. *The number of included RCTs against the total number of RCTs indexed in the PubMed database for the study period (1966–2018). A year 1993 and 1998 are marked with vertical dashed lines.*

To address these relevant issues, we have included the additional analyses in the Methods and Results section. In our discussion, we now write: *"First, our selection of RCTs may have been biased as full-text RCTs may not have been missing at random (e.g. with particular year of publication or RCT methodology). Most RCTs are not included as we do not have access to full-text data, and this makes it difficult to make definitive conclusions on the missing RCTs with regard to risk of bias, CONSORT statement use, and trial registration. However, an additional analysis of over 7000 RCTs with an uncertain year of publication (7,307) that were excluded yielded similar distribution of the risk-of-bias domain probabilities. Moreover, the included RCTs in our analyses were generally in line with the overall number of potential RCTs in the PubMed database (Suppl. Figure S6), except for the time period between 1993 and 1998 when libraries switched from scanned versions to online subscriptions, potentially lowering the yield of automated full text downloads. However, this period is before the release and implementation of the discussed guidelines, and our sample still maps the important patterns in the overall RCT publications over time."*

2. *Automatic extraction of information from meta-data is at the core of the study, and it would have been virtually impossible to check all this by hand. But the algorithms used are imperfect (not that I claim humans would be perfect in determining the risk of bias, for instance). Some years ago, I have participated in a project where we used genderize, for instance, and results are not perfect. Was there an attempt to manually check the automated results on a subsample? Otherwise, some presentation of the performance of the algorithm and the possible impact on the results may be useful. For RoB, the agreement of the ML algorithm with humans is reported to be 71% (or 65%, different figures and different references being used in the methods and discussion, which is confusing). What impact could this have on the results? In particular, do the confidence intervals reported account for possible misclassification?*

Answer: This is an excellent suggestion. We have now validated our predictions with a newly designed validation dataset comprised of human expert ratings of RCTs. This has allowed us to determine the robustness of our results, including which RoB score is best and which is worst. Our manual validation based on 63,327 extracted and standardized human risk-of-bias assessments, as published in a Cochrane Database of Systematic Reviews showed accuracies of over 70% and kappa values between 0.25–0.43. Even though this sounds far from impressive, it should be borne in mind that agreement between human reviewers is often not much higher. For example, based on 376 RCTs overall kappa values of interrater risk-of-bias scores ranged from 0.40–0.42, although some domains were clearly higher, such as random sequence bias agreements between human raters (kappa 95% CI: 0.56–0.71).

We now write in the Methods section:

"Risk-of-bias assessment validation
To determine the accuracy of the automated risk of bias assignment to RCTs in our database, we validated a large subset of RCTs with human assessments obtained from systematic reviews. To this end, we inspected all PMIDs of RCTs included in the 8,075 systematic reviews (Issue 6, 2020) published in the Cochrane Database of Systematic Reviews (CDSR). The CDSR is part of the international charitable Cochrane organization, with more than fifty review groups based at research institutions worldwide, aiming to facilitate evidence-based choices about health interventions. Contributions to the database come from these review groups as well as from ad hoc teams. The systematic reviews in the CDSR were identified through PubMed, for the period to 2000 and 2020. This was done with the NCBI's EUtils API with the following query: "Cochrane Database Syst Rev"[journal] AND ("2000/01/01"[PDAT]: "2020/05/31"[PDAT])". We limited assessment of the latest systematic review updates as to prevent overlap between included RCTs. Review protocols were excluded.

All systematic review tables containing the words 'bias' and 'risk' were inspected on human export risk-of-bias text associated with a PMID. If a PMID matched with a PMID in our full-text database and the risk of bias domain concerned 'allocation concealment', 'random sequence generation', 'blinding of participants and personnel' or 'blinding of the outcome', the human risk-of-bias text was extracted.

Since the risk-of-bias data reporting in the CDSR is not imposed or standardized. We, therefore, manually inspected all human risk-of-bias texts and assigned the judgment to the correct and standardized risk-of-bias category. Judgments were binarized into 'low' and 'high/unclear' risk of bias and used to validate our automated binarized risk-of-bias probabilities

(i.e., <= 0.5 'low' risk, and > 0.5 'high/unclear' risk) in terms of accuracy, sensitivity, specificity, and kappa."

In the Results section:

"Risk-of-bias assessment validation
*In total, 63,327 matching human risk-of-bias judgments and automated risk-of-bias predictions were extracted from 17,394 unique RCTs included in a systematic review (**Table 1**). Overall, automated accuracy to determine 'randomization bias', 'allocation bias' and 'blinding of people bias' was above 70%. The 'blinding of outcome bias' had a lower accuracy, namely 63.3%. The distribution of risk-of-bias predictions are shown in **Supplementary Figure S5**.*

*We also classified the chance-corrected level of agreement in terms of kappa. Kappa ranges from: 'poor' (<0.00), 'slight' (0.00–0.20), 'fair' (0.21–0.40), 'moderate' (0.41–0.60), 'substantial' (0.61–0.80), to 'almost perfect' (0.81–1.0). Within this scale, moderate agreement was found for randomization and blinding of people bias and fair agreement was found for allocation bias and blinding of outcome bias (**Table 1**)."*

And in the Discussion:
"Our manual validation based on 63,327 extracted and standardized human risk-of-bias assessments, as published in a Cochrane Database of Systematic Reviews showed accuracies of over 70% and kappa values between 0.25–0.43. Even though this sounds far from impressive, it should be borne in mind that agreement between human reviewers is often not much higher. For example, based on 376 RCTs overall kappa values of interrater risk-of-bias scores ranged from 0.40–0.42, although some domains were clearly higher, such as random sequence bias agreements between human raters (kappa 95% CI: 0.56–0.71)."

3. *The number of publications has been increasing over years, and as a mechanical consequence, the citations also. The average impact factors of journals has also increase, as well as the proportion of journals indexed in the Journal Citation Reports with IF > 10, for instance. More precisely, 1.1% of all journals listed in JCR (SCIE) in 199 had IF > 10, 1.7% in 2009 and 3.0% in 2019. Those proportions may have been quite higher in the journals considered in the review (but it's only a guess). As a result, why not use a relative IF cut-off (e.g. top 10% or top 5%), instead of using a fixed IF cut-off (≤10 vs >10)? Also, the table S2 lists journals classified as with IF > 10 for all individual publications in the dataset, but for instance Leukemia's IF only reached 10 or more in 2012. Similarly, for Annals of Oncology it was in 2016. It is striking that no RCT would be included for these journals before those years. Perhaps another IF that the one of JCR (Clarivate Analytics) was sued, but this should be specified.*

Answer: We thank the reviewer for the interesting suggestion. However, we have already presented JIFs with different cutoffs: We used a JIF threshold of 10 as the primary cutoff based on JIF distributions (see Supplementary Table S2 and Supplementary Figure S1) and previous evidence for sensitivity to assess RCT quality using this cutoff [6]. We also performed sensitivity analyses for JIF cutoff thresholds at 3 and 5, yielding similar results The JIF is in general not a reliable indicator, and we have refrained of performing additional analyses with different cutoffs or relative JUF cutoffs.

4. *The average increase of citations may also explain why the h-index of authors also increased. It is therefore difficult to disentangle trends specific to RCTs to global trends of the whole medical literature. Asking for a specific answer to this point would be unfair to some extent, because it may require a large amount of work, but from a scientific point of view, the question of whether the h-index of authors increased more for RCTs than for other types of studies seems more interesting that simply describing the evolution of the h-index. That said, I wonder whether this any question about the evolution if the h-index of authors is really relevant and useful in the present study, which is not about the evolution of the citation network among authors of the biomedical literature.*

Answer: We agree with the reviewer and have removed the use of H-index of the main analyses which are now part of the supplement.

5. *Risk of bias is not a binary concept in Cochrane's evaluation. The RoB2 tool classifies risk of bias judgments as low risk of bias, some concerns, and high risk of bias. What was considered as "risk of bias" in the study, "high risk of bias"? More clarity would be welcome.*

Answer: We agree, that the RoB2 has different and better output labels compared to RoB1. The category 'some concerns' is only available in RoB2. However, RoB2 scores are only operational in studies published in the last year (https://doi.org/10.1002/9781119536604.ch8, Higgins et al, 2019), so not included in our study. We operationalized the risk-of-bias as 'low risk' versus 'high or unclear risk'. The probability below 0.5 corresponds to the 'low bias' assessments. Probabilities above 0.5 to 'high/unclear' judgements. We have also used this threshold to binarize our probabilities in the validation characterization. We have kept the probabilities in the other plots to prevent information loss due to binarization.

We have now specified this in the Methods section: *"The machine-learning output is a probability, with values below 0.5 corresponding with 'low risk', and above 0.5 with 'high or unclear' risk of bias."*

6. *Additional comments*

- *It is unclear in the abstract what characteristics of publication and authors are, and how they would relate to the main issue which is about methodological quality and bias.*

Answer: We agree with the reviewer, and have substantially changed the abstract to specify the characteristics of publication and authors, even though character limits prevents us from detailed descriptions.

- *I agree with problems of reports of RCTs with randomization, allocation concealment, and blinding but is difficult to ascertain what is the true treatment effect, in particular because this would need to formally define the target population. I would therefore recommend softening the assertion "the majority of RCT findings have inflated estimates" because this is not really supported by strong evidence.*

Answer: We agree with the reviewer and have changed the wording.

- *The (expected) benefit of registration could be better explained, instead of simply noting it has been made mandatory by IMCJE.*

Answer: We thank the reviewer for the suggestion, and have expanded the text on the ICMJE registration benefits.

- *I acknowledge that wording can become extremely tedious when describing results, but writing "The risk of bias in allocation concealment was consistently lower in trials published in journals with JIF larger than 10" implies that the risk of bias in allocation concealment in a quantity, when in fact what is lower is the proportion of trials with low (?) risk of bias.*

Answer: This is a fair point, and we have changed the text here and carefully read the text for other instances.

- *The link of reference 15 (protocol) does not work, but the following one does: "https://eur04.safelinks.protection.outlook.com/?url=https%3A%2F%2Fosf.io%2F27f53%2F%23&data=04%7C01%7Cc.vinkers%40amsterdamumc.nl%7C511d6afe7d0d41858ed708d880e54dee%7C68dfab1a11bb4cc6beb528d756984fb6%7C0%7C1%7C637401069631408525%7CUnknown%7CTWFpbGZsb3d8eyJWIjoiMC4wLjAwMDAiLCJQIjoiV2luMzIiLCJBTiI6Ik1haWwiLCJXVCI6Mn0%3D%7C2000&sdata=%2BBMNwU6ZkoivOFHBxpgBaxFKMXrLxo157jerxJjNGxc%3D&reserved=0!". Please consider revising.*

Answer: We have changed this as it lacked a '.'.

==

Reviewer #4:

Thanks for giving me the opportunity to review this manuscript, which explores quality of RCTs published between 1966 and 2018. The authors used machine learning techniques to extract information on study characteristics, risk of bias, trial registration, CONSORT statements, H-indexes. They found that overall quality of RCTs improved over time (lower bias, more CONSORT reporting and more study registrations), but a considerable amount of RCTs still lacks basic quality characteristics.

Answer: We would like to thank the reviewer for his/her constructive comments. We have addressed each comment and have indicated how changes have been incorporated in our revised manuscript.

1. *Introduction, line 89-91: The authors state that there is already a study on RCT quality that included more than 20,000 RCTs from Cochrane reviews. This seems to be a high number of RCTs, but the authors further state that "large-scale evidence (…) is lacking". It would be helpful to know as to why reference 14 does not provide enough evidence on time trends (i.e. bias as only RCTs from Cochrane reviews were included).*

Answer: Dechartres et al describe RCTs included in all Cochrane reviews published between March 2011 and September 2014 reporting an evaluation of the Cochrane risk of bias items. This is thus not a total sample of RCTs, but a selected sample, both in quality and scope, assessment, and year published (as they were part of Cochrane reviews between 2011 and

2014). The publication is a solid basis for our work, but cannot be readily extrapolated to the 176,620 RCTs currently analyzed.

To reflect these differences, we now indicate in the Introduction: "Nevertheless, *based on 20,920 RCTs from Cochrane reviews published between 2011 and 2014, there are indications that poor reporting and inadequate methods have decreased over time [6]. However, unbiased large-scale evidence on trends of RCT characteristics and methodological quality across medical disciplines over time is currently lacking."*

2. *Methods, line 128: The authors report on a human-RobotReviewer agreement of 65% on average, stating that this is similar to 79% human-human agreement. It would be helpful if the authors provided reasons as to why this difference is irrelevant or does not impact validity of results*

Answer: We thank the reviewer and agree that additional analyses were need with regard to human-RobotReviewer agreements. We have now validated our predictions with a newly designed validation dataset comprised of human expert ratings of RCTs. This has allowed us to determine the robustness of our results, including which RoB score is best and which is worst. Our manual validation based on 63,327 extracted and standardized human risk-of-bias assessments, as published in a Cochrane Database of Systematic Reviews showed accuracies of over 70% and kappa values between 0.25–0.43. Even though this sounds far from impressive, it should be borne in mind that agreement between human reviewers is often not much higher. For example, based on 376 RCTs overall kappa values of interrater risk-of-bias scores ranged from 0.40–0.42, although some domains were clearly higher, such as random sequence bias agreements between human raters (kappa 95% CI: 0.56–0.71).

We now write in the Methods section:

"Risk-of-bias assessment validation

To determine the accuracy of the automated risk of bias assignment to RCTs in our database, we validated a large subset of RCTs with human assessments obtained from systematic reviews. To this end, we inspected all PMIDs of RCTs included in the 8,075 systematic reviews (Issue 6, 2020) published in the Cochrane Database of Systematic Reviews (CDSR). The CDSR is part of the international charitable Cochrane organization, with more than fifty review groups based at research institutions worldwide, aiming to facilitate evidence-based choices about health interventions. Contributions to the database come from these review groups as well as from ad hoc teams. The systematic reviews in the CDSR were identified through PubMed, for the period to 2000 and 2020. This was done with the NCBI's EUtils API with the following query: "Cochrane Database Syst Rev"[journal] AND ("2000/01/01"[PDAT]: "2020/05/31"[PDAT])". We limited assessment of the latest systematic review updates as to prevent overlap between included RCTs. Review protocols were excluded.

All systematic review tables containing the words 'bias' and 'risk' were inspected on human export risk-of-bias text associated with a PMID. If a PMID matched with a PMID in our full-text database and the risk of bias domain concerned 'allocation concealment', 'random sequence generation', 'blinding of participants and personnel' or 'blinding of the outcome', the human risk-of-bias text was extracted.

Since the risk-of-bias data reporting in the CDSR is not imposed or standardized. We, therefore, manually inspected all human risk-of-bias texts and assigned the judgment to the correct and standardized risk-of-bias category. Judgments were binarized into 'low' and

'high/unclear' risk of bias and used to validate our automated binarized risk-of-bias probabilities (i.e., <= 0.5 'low' risk, and > 0.5 'high/unclear' risk) in terms of accuracy, sensitivity, specificity, and kappa."

And in the Results section:

"Risk-of-bias assessment validation
*In total, 63,327 matching human risk-of-bias judgments and automated risk-of-bias predictions were extracted from 17,394 unique RCTs included in a systematic review (**Table 1**). Overall, automated accuracy to determine 'randomization bias', 'allocation bias' and 'blinding of people bias' was above 70%. The 'blinding of outcome bias' had a lower accuracy, namely 63.3%. The distribution of risk-of-bias predictions are shown in **Supplementary Figure S5**.*

*We also classified the chance-corrected level of agreement in terms of kappa. Kappa ranges from: 'poor' (<0.00), 'slight' (0.00–0.20), 'fair' (0.21–0.40), 'moderate' (0.41–0.60), 'substantial' (0.61–0.80), to 'almost perfect' (0.81–1.0). Within this scale, moderate agreement was found for randomization and blinding of people bias and fair agreement was found for allocation bias and blinding of outcome bias (**Table 1**)."*

And in the Discussion:

"Our manual validation based on 63,327 extracted and standardized human risk-of-bias assessments, as published in a Cochrane Database of Systematic Reviews showed accuracies of over 70% and kappa values between 0.25–0.43. Even though this sounds far from impressive, it should be borne in mind that agreement between human reviewers is often not much higher. For example, based on 376 RCTs overall kappa values of interrater risk-of-bias scores ranged from 0.40–0.42, although some domains were clearly higher, such as random sequence bias agreements between human raters (kappa 95% CI: 0.56–0.71)."

3. *Methods, line 160, 161: The authors state that regression analysis was used, but it would be nice if the authors further explained details of the regression model.*

Answer: We used two types of linear regression. Standard regression for the continuous independent variables (i.e., risk-of-bias scores) and logistic regression for dichotomous independent variables (i.e., CONSORT Statement mentioned and presence of RCT registration). Each model contained an intercept and 'time'. Time which was modelled as a factor, with an estimate for each time period. Models were fitted with the standard glm function in R ('gaussian' and 'binomial' link distributions for the standard and logistic regressions, respectively). Post-hoc comparisons between time period was determined with Tukey's HSD post-hoc test (R package 'multcomp') [7].

4. *Methods, line 118-120: Why did the authors summarize all articles form 2010 to 2018, when in the methods it is stated that 5-year periods were considered (plus prior 1990 in one stratum)?*

Answer: Due to our specific odd time-period and little to no effect on the model's output with various binnings, we decided to keep the thresholds between bins at the 5 years levels. This is more appealing graphically as well (i.e., "2000-2005" rather than, for example in a equalized spacing, "2001.3 – 2004.7" in the x-labels). Our last bin lacks therefore some time. As we are

not interested in strict binary changes, we based all calculations on the proportion, rather than the total. Therefore, all RCT data is normalized for its specific time-period/bin, keeping the overall pattern unaffected.

5. *Results, line 171: Full-text was only available for 60% of identified RCTs. I would suggest that the authors elaborate on potential risks this imposes on the results.*

Answer: We agree that a proportion of identified RCTs could not be analyzed. We have performed additional analyses regarding missing RCTs. Most RCTs are not included as we do not have access to full-text data. This makes it difficult to make definitive conclusions on the missing data with regard to risk of bias, CONSORT statement and trial registration. However, we did exclude RCT publications with an uncertain year of publication (7,307). Therefore, we compared these 7,307 RCT with the included 176,620 RCTs. The distribution of the risk-of-bias domain probabilities of included and excluded RCTs were comparable, with very similar interquartile ranges (see Suppl. Table S2). Moreover, the presence of the 'With CONSORT Statement' and 'RCT Registration' outcomes were 3–4% lower in the excluded RCTs (Suppl. Table S3). Moreover, the full text sample size was generally in line with the overall number of potential RCTs in the PubMed database (Suppl. Figure S6). Generally, the % of included RCTs was stable over time, except for the time period between 1993 and 1998 when the library switched from scanned versions to online subscriptions, potentially lowering the yield of automated full text downloads. this period is before the release and implementation of the discussed guidelines, we think our sample still maps the important patterns in the overall RCT population. We have added these results to the manuscript and mentioned the limitation in the Discussion.

To address these relevant issues, we have included the additional analyses in the Methods and Results section. In our discussion, we now write: *"First, our selection of RCTs may have been biased as full-text RCTs may not have been missing at random (e.g. with particular year of publication or RCT methodology). Most RCTs are not included as we do not have access to full-text data, and this makes it difficult to make definitive conclusions on the missing RCTs with regard to risk of bias, CONSORT statement use, and trial registration. However, an additional analysis of over 7000 RCTs with an uncertain year of publication (7,307) that were excluded yielded similar distribution of the risk-of-bias domain probabilities. Moreover, the included RCTs in our analyses were generally in line with the overall number of potential RCTs in the PubMed database (Suppl. Figure S6), except for the time period between 1993 and 1998 when libraries switched from scanned versions to online subscriptions, potentially lowering the yield of automated full text downloads. However, this period is before the release and implementation of the discussed guidelines, and our sample still maps the important patterns in the overall RCT publications over time."*

6. *Discussion, line 291, 292: "Additionally, making data sets available according to the FAIR principles arguably will improve the situation". It would be helpful if the authors gave reasons as to why data availability was not included in the assessment criteria for RCTs.*

Answer: This was, to the best of our knowledge, not possible to obtain in an automated fashion.

7. *Minor issues:*

- *Introduction, line 81: The authors mention the Hong Kong Principles without further elaboration. It would be helpful to readers if the authors provided a short (i.e. one sentence or a relative clause) description of these principles.*

Answer: We have now expanded the text to elaborate on the Hong Kong Principles.

- *Figure 1: The authors should report all numbers as "n=" (also the subcategories that were excluded).*

Answer: We have harmonized the flow chart. The excluded set of >7,307 RCTs is used for a comparative analysis and added to the flow chart.

- *Figure 4: Please check the caption (drop "plotted over time")*

Answer: We have corrected this, thank you.

- *Discussion, lines 302, 303: The authors could provide reasons why one medical field had higher RCT quality than others (i.e. more pragmatic RCTs in one area than in the other).*

Answer: This is a fair point. We have expanded the text.

References

1. Damen JA, Lamberink HJ, Tijdink JK, Otte WM, Vinkers CH, et al. (2018) Predicting questionable research practices in randomized clinical trials. Open Science Framework, https://osf.io/27f53/ December 19th, 2018.
2. Kousta S, Ferguson C, Ganley E (2016) Meta-Research: Broadening the Scope of PLOS Biology. PLoS Biol 14: e1002334.
3. Begg C, Cho M, Eastwood S, Horton R, Moher D, et al. (1996) Improving the quality of reporting of randomized controlled trials. The CONSORT statement. JAMA 276: 637-639.
4. Zwierzyna M, Davies M, Hingorani AD, Hunter J (2018) Clinical trial design and dissemination: comprehensive analysis of clinicaltrials.gov and PubMed data since 2005. BMJ 361: k2130.
5. Schulz KF, Altman DG, Moher D, Group C (2010) CONSORT 2010 statement: updated guidelines for reporting parallel group randomised trials. BMJ 340: c332.
6. Dechartres A, Trinquart L, Atal I, Moher D, Dickersin K, et al. (2017) Evolution of poor reporting and inadequate methods over time in 20 920 randomised controlled trials included in Cochrane reviews: research on research study. BMJ 357: j2490.
7. Hothorn T, Bretz F, Westfall P (2008) Simultaneous inference in general parametric models. Biom J 50: 346-363.
