# Supplementary material for: The methodological quality of 176,620 randomized controlled trials published between 1966 and 2018 reveals a positive trend but also an urgent need for improvement
Source: PLoS Biol. 2021 Apr 19;19(4):e3001162. doi: 10.1371/journal.pbio.3001162 (PMC8084332; doi:10.1371/journal.pbio.3001162)
Supplement: Attachment — Submitted filename: Rebuttal.docx [file pbio.3001162.s016.docx]

**Academic Editor**

*Based on the reviews, we will probably accept this manuscript for publication, assuming that you will modify the manuscript to address the remaining points raised by the reviewers. Please also make sure to address the data and other policy-related requests noted at the end of this email.*

**Answer:** We would like to thank the editor for his constructive comments. We have addressed all comments and have indicated how changes have been made.

*a) Please attend to the remaining requests from reviewer #1.*

**Answer:** Please see below for the responses to the remaining requests.

*b) Please re-word your Abstract to fit the verbose style that we use at PLOS Biology (not the current structured format that is more typical of clinical journals).*

**Answer:** We have now changed this.

*c) The current title is rather cumbersome and repetitive. Please change it to something more appealing; we suggest: "**Analysis of the methodological quality of 176,620 randomized controlled trials published between 1966 and 2018 reveals a positive trend but urgent need for improvement."*

**Answer:** We agree and we have now changed this (we have changed your valuable suggestion a bit).

*d) Please attend to my Data Policy requests below.*

**Answer:** We have generated a publicly available release with all scripts and data used to generate the figures and tables of the current manuscript, which is available at <https://zenodo.org/record/4560756#.YDdOF3nvKUl> (DOI: 10.5281/zenodo.4560756), and we have included this in the manuscript.

Under the heading *Data sharing*, we now state: “All data including code and risk-of-bias/human validation data are available at <https://github.com/wmotte/RCTQuality> or <https://doi.org/10.5281/zenodo.4362238>)”

With regard to supplemental information, we will change this as well.

**Reviewer #1:**

*I would like to thank the authors for having answered my comments and modified the manuscript.
I have some additional comments.*

**Answer:** We would like to thank the reviewer for his/her constructive comments. We have addressed each comment and have indicated how changes have been incorporated in our revised manuscript.

*1. Objective: I would remove the word "unbiased": how do the authors can be sure that their evaluation is unbiased.*

**Answer:** We agree. We initially used the word unbiased to emphasize the automatic classification, rather than manual labeling. We have removed it from the current manuscript.

*2. In the abstract, the sentence is not really clear: "we mapped RoB trends over time in RCT publications including journal and author characteristics". The end of the sentence is not clear, journal and author characteristics are not part of RoB*

**Answer:** We agree. We changed ‘including’ to ‘in relation to’.

*3. I really thank the authors for having conducted a validation with RoB assessment reported in Cochrane reviews. The interest of using Cochrane reviews that could be highlighted is that RoB is evaluated the same way in all Cochrane reviews using the RoB tool by trained reviewers.*

**Answer:** We thank the reviewer for the positive note. We agree and how now included the following sentence:

*“Moreover, we validated standardized human risk-of-bias judgments by trained reviewers from Cochrane systematic reviews with automated risk-of-bias predictions (Table 1).”*

*4. How do the authors define the parameter accuracy?*

**Answer:** We have added this to the manuscript: “The accuracy is expressed as the proportion of those RCTs correctly categorized by the model, namely as (true positives + true negatives) / (true positives + false positives + false negatives + true negatives).”

*5. Which RoB did they take for trials included in two Cochrane reviews? Did this situation happen?*
**Answer:** If more than one systematic review reports a risk-of-bias score, we included all variants. This did indeed occur.

*6. The Kappa values are very low and lower than the Kappa values between reviewers assessing risk of bias using the Risk of bias tool (there is an abundant literature on the topic).*

**Answer:** Classification of all risk-of-bias domains is a difficult task, both for humans and also for software tools. We refer to human kappa values (ref. 30), which are as low as 0.4 for some domains. The low agreement between human raters is precisely the reason why the risk-of-bias tool was recently revised into version 2 (E.g., BMJ 2019; 366 doi: https://doi.org/10.1136/bmj.l4898). Too many human raters rated risk-of-bias domains differently. This relative high variability and imperfect accuracy is particularly problematic for individual trial characterization. During the process of meta-analyses within the context of systematic review conduct a trial will be included or excluded in an aggregated analysis based on whether the risk-of-bias is high or not. Meta-analyses typically have relative few studies (i.e., 3–15). Consequently, the inclusion or exclusion of an individual trial due to incorrect risk assessment will have a large impact. In our study, however, we used the risk-of-bias characterization differently. We are not focusing on individual trials, but we want to know how patterns in risk-of-bias are distributed. These patterns are affected by noise, noise included from study selection as well as imperfect risk-of-bias classification. However, the patterns underlying this noise are nonetheless clearly visible (and explainable, as thousands of RCTs are averaged out. Despite the imperfect model performance, the overall patterns do hold at a group level. We also think that the observed patterns are explainable, given the improvements over time. The validation numbers will hopefully prevent users to rely too much on automatic classification of individual trials, as well as stimulate improvements of the current risk-of-bias classification algorithms, of which we used the most accurate at the moment.

We have now included several sentences of this important topic in the Discussion section of the manuscript.

*“Second, the risk of bias is inherently difficult to assess reliably. Experts’ assessments of trials show that labeling the same trials for different Cochrane reviews resulted in substantial differences [30, 31]. Probabilities assigned with machine learning are based on a large set of human-assigned labels, and a direct comparison shows computerized assessment performance of 71.0% agreement [32].* *Our manual validation based on 63,327 extracted and standardized human risk-of-bias assessments, as published in a Cochrane Database of Systematic Reviews showed accuracies of over 70% and kappa values between 0.25–0.43. Even though this sounds far from impressive, it should be borne in mind that agreement between human reviewers is often not much higher. Classification of risk-of-bias domains is a difficult task, both for humans and automated software. For example, based on 376 RCTs overall kappa values of interrater risk-of-bias predictions ranged from 0.40–0.42, although some domains were clearly higher, such as random sequence bias agreements between human raters (kappa 95% CI: 0.56–0.71) [30]. The low agreement between human raters is precisely the reason why the risk-of-bias tool was recently revised into version 2 [33]. The relative high variability and imperfect accuracy is particularly problematic for individual trial characterization where the inclusion or exclusion of an individual trial due to incorrect risk assessment will have a large impact. In our study, however, we applied the risk-of-bias characterization differently, and did not focus on individual trials but rather studied patterns in risk-of-bias distributions.”*

*7. On which human reports and on how many are based algorithms of RobotReviewer regarding risk of bias?*

**Answer:** RobotReviewer used a distinct dataset of crowd-labelled data. This crowd-data is not publicly available and is constantly being updated (see: http://crowd.cochrane.org/index.php). The authors of RobotReviewer matched the full text of 12,808 PDFs with these labels to build their risk-of-bias tool. See: Marshall IJ, et al. J Am Med Inform Assoc 2016;23:193–201, doi:10.1093/jamia/ocv044 for details.

*8. I don't understand when the authors say that "RoB data reported in CDSR not imposed or standardized", it is not true. The RoB judgment is almost always expressed the same way in high, low or unclear (or yes, no, unclear) as it is the format used in Revman. It is possible to extract data from the Cochrane review as an xml file, and to directly extract the RoB table with the judgment. I don't know if this is what the authors did.*
**Answer:** We apologize for the confusion. In theory the labels should be equal, however there are inconsistencies – in particular in the older systematic review data – in capitalization. The data is also often extended with study-specific details. For example: “low (nurses)” in blinding of personnel risk outcome – in this case nurses – or in the labels itself (e.g., “Outcome reporting: blood pressure”). This was unexpected to us as well, therefore we manually checked and standardized the entries. Because this may indeed be confusing and relates to very specific pre-processing details, we have changed the phrasing to the more general: “All human risk-of-bias texts and assigned judgment were manually inspected and assigned to the proper risk-of-bias category.”

*9. In the statistical analysis, I don't understand why the authors reported either p-values for trends or p-values for comparisons across two categories. It is not consistent throughout the manuscript. They reported trend tests for RoB but not for publication characteristics. Why? Trend tests could be used.*

**Answer:** We have now added trend results for the publication characteristics as well.

*10. In the results, it is not clear from the abstract whether the percentages refer to the first and last time categories or not (first and last decades) and for the first time category, is it <1990 or 1990-1995? I am a bit lost with the time categories. Looking at the text, the oldest category seems to be 1990-1995, once <1990 is reported in text (why?) but looking at the Figures, <1990 is systematically reported. I think it would be better to be consistent throughout. Either the authors consider that trials published before 1990 are too old and they exclude them, or if not, they have to use the category <1990 as the reference.*

**Answer:** We removed the single reference to ‘<1990’ from the Results section. We do think it is informative to show the data of the rather elongated 1966–1990 period. However, for the statistical comparison we have chosen to use the 1990-1995 category as the baseline.

We also now explicitly explain the rationale in Methods section: *“Reported P-values correspond to the overall trend estimate or the comparison of the average value per year in the 1990–1995 and 2010–2018 strata obtained from posthoc Tukey corrected comparisons. This post-1990 period was chosen to cover the first years following significant awareness on the need to report transparently, in comparison to the latest years in our dataset.”*

*11. In the Discussion, the summary of main findings puts a major emphasis on author characteristics including women while only a few results are presented on this part. This should be consistent.*

**Answer:** We have modified the summary of the Discussion to reflect the general patterns more clearly.

*12. Many authors do not report registration number in trial publication but this does not mean that these trials were not registered (I agree that it is important to report the registration number in the publication). In contrast, it is not because you don't mention Consort in text that you do not comply with. Therefore I suggest the authors to be a little bit more careful in wording*

**Answer:** We agree and changed the discussions accordingly: *“Moreover, despite the requirement of trial registration for publication since 2004, still in 2017 a substantial percentage of published RCTs do not report registration numbers. This suggests a lack of registration in a subset of RCT, although the absence of registration numbers does not necessarily imply absence of registration.”*

and:

*“Furthermore, many RCTs do not mention the CONSORT guidelines in their full text, and more so for journals with lower impact factors. Despite the accessibility of reporting guidelines, researchers are generally not required to adhere to them, and, more problematic, requirements are not strictly enforced and non-compliance to all the items on the reporting guideline is not sanctioned [**4**,**27**]. It is important to note that not mentioning CONSORT does not necessarily imply non-transparent reporting, although explicit mentioning is preferable.”*

*13. The term score is still mentioned*

**Answer:** We have changed ‘score’ to ‘prediction’ in the manuscript – apologies.

*14. To evaluate the item incomplete outcome data, it is not necessary to rely on trial registration. Access to trial registration is mainly important for selective outcome reporting.*

**Answer:** We agree and have changed this in the Discussion.

*15. Table 1: I would present 95% CI everywhere and I would take the usual formulation for Rob items*

**Answer:** We have added 95% CIs to all outcomes, rather than only to the ‘accuracy’ (i.e., to the sensitivity, specificity and kappa values). Also, the formulations have been adjusted in Table 1 to the usual formulations.

**Reviewer #4:**

*I would like to congratulate the authors for the improvement. All issues have been addressed adequately.*

**Answer:** We would like to thank the reviewer for his/her positive assessment.
